# Development of In Vitro Assays with the Canine Hookworm *Uncinaria stenocephala* and Assessment of Natural Plant Products for Anti-Parasitic Activity

**DOI:** 10.3390/pathogens12040536

**Published:** 2023-03-29

**Authors:** Heidi A. Geisshirt, Charlotte S. Bonde, Caroline Marcussen, Helena Mejer, Andrew R. Williams

**Affiliations:** Department of Veterinary and Animal Sciences, University of Copenhagen, DK-1870 Frederiksberg, Denmark

**Keywords:** hookworms, *Uncinaria stenocephala*, seaweed, in vitro, anthelmintic

## Abstract

Enteric helminth infection is an increasing concern in companion animals due to reports of resistance to commonly used anthelmintic drugs. Thus, the assessment of new therapeutic options such as bioactive dietary additives is of high importance. Here, we adapted egg hatch, larval migration, and larval motility assays to screen extracts of several natural ingredients against the canine hookworm *Uncinaria stenocephala*, a prevalent parasite of dogs in northern Europe. Egg hatch and larval migration assays were established showing that the anthelmintic drugs levamisole and albendazole had strong anti-parasitic activity against *U. stenocephala*, validating the use of these assays for the assessment of novel anti-parasitic substances. Subsequently, we identified that extracts from the seaweed *Saccharina latissima*, but not extracts from grape seed or chicory, significantly inhibited both hatching and larval migration. Finally, we showed that α-linolenic acid, a putative anti-parasitic compound from *S. latissima*, also exhibited anti-parasitic activity. Collectively, our results established a platform for the screening for anthelmintic resistance or novel drug candidates against *U. stenocephala* and highlighted the potential use of seaweed extracts as a functional food component to help control hookworm infection in dogs.

## 1. Introduction

Intestinal parasites are widespread in animals, both livestock and pets [1,2,3]. Enteric parasite infection may cause growth retardation and/or diarrhea, and in some cases, zoonotic infections may be transmitted between animals and humans [4]. Parasitic worms (helminths) from the ascarid and ancylostomatid (including hookworms) groups are some of the most common parasites to infect cats and dogs on a global scale [5,6]. The routine use of broad-spectrum anthelmintics has meant that these parasites are not always considered a major problem. However, in livestock, anthelmintic resistance is widespread in sheep, cattle, and horses, and the problem seems to be increasing [7,8]. Notably, multi-drug anthelmintic resistance has also now been detected in canine and feline helminths, including hookworms, and is possibly underestimated and spreading among dogs [9,10,11].

Human hookworm infections are a neglected tropical disease, infecting millions of people globally, primarily in rural communities, and they have a major impact on health and livelihoods [12,13,14]. Similarly, in dogs hookworms may cause iron deficiency anemia and poor growth, depending on the severity of infection and the species [15,16]. Moreover, they may also pose a considerable risk to human health, as some species are zoonotic (e.g., *Ancylostoma* spp.) and may even cause patent infections in humans [6,17]. The northern canine hookworm, *Uncinaria stenocephala*, is a parasite of dogs in temperate areas which can have a medium-high prevalence [18,19,20]. Whilst considered less pathogenic than *Ancylsotoma caninum*, this parasite can still cause clinical disease. Importantly, *U. stenocephala* appears to be more refractory to anthelmintic drug treatment than other canine hookworms [21,22], emphasizing the continuing need for research on novel control options for this parasite.

In livestock, high levels of anthelmintic drug resistance have meant that novel control and treatment (i.e., alternatives to synthetic drugs) options have been increasingly explored. One of these options is the use of plant extracts or other bioactive dietary compounds as functional food components [23,24,25,26,27]. Bioactive plants have been used for centuries but only recently have controlled scientific studies been employed to validate the use of these complementary treatment options and to identify the active compounds. Examples of bioactive plants with demonstrated bioactivity against parasitic nematodes include chicory, tannin-containing plants such as sainfoin, and marine macroalgae such as seaweed [24,25,28,29]. Bioactive compounds within these plant sources may exert anthelmintic activity through exerting pharmacological-like actions against nematodes which can be mimicked in in vitro assays [28].

Given the rising concerns regarding anthelmintic drug resistance in companion animals, and a wider appreciation of the drawbacks of using chemotherapy to treat infections in animals (e.g., chemical residues in the environment), there has been an increasing interest in the development of functional food components (e.g., phytochemicals) in pet foods that may improve gut health [30,31]. To this end, in the present study we explored whether natural plant-derived compounds may represent a novel treatment option for *U. stenocephala*. We first adapted in vitro anti-parasitic assays that have been described for other helminth species of *U. stenocephala* [32,33,34]. We subsequently applied these assays to test six different natural substances for activity against this parasite.

## 2. Materials and Methods

### 2.1. Chemicals and Plant Extracts

An overview of the tested plant extracts and compounds is provided in Table 1. Levamisole, albendazole, trans-cinnamaldehyde, dimethyl sulfoxide (DMSO), RPMI-1640 media and α-linolenic acid were obtained from Sigma-Aldrich (Stellenborsch, Germany). Grape seed extract, consisting of >95% condensed tannins [35], was purchased from Bulk Powders (Colchester, UK). A chicory (*Cichorium intybus*) extract (cv. Spadona) enriched in sesquiterpene lactones was prepared as previously described [36]. Four different seaweed extracts were produced as described by Bonde et al. [24]. Briefly, *Saccharina latissma* was sourced from either Grenå, Denmark, or the Faroe Islands. From each source location, extracts were prepared using water and methanol (polar extracts; SW1, SW2), or dichrolormethane and methanol (non-polar extracts; SW3, SW4). The chemical composition of the extracts has previously been reported [24].

### 2.2. Parasites

Fecal material was collected from a dog kennel with a known history of *U. stenocephala* infections. Samples were collected following natural defecation in the morning and cooled for transport to the laboratory. Two-gram fecal aliquots were used for determining the number of parasite eggs in each fecal sample, using a concentration McMaster technique [37] with a lower detection limit of 20 eggs per gram of feces (EPG). The parasite species of the eggs was estimated based on egg morphology and size. The eggs of *U. stenocephala* (72 − 92 × 37 − 55 μm) are on average larger than the eggs of *Ancylostoma caninum* (55 − 74 × 37 − 43 μm), allowing a reasonable degree separation between *U. stenocephala* and *Ancylostoma* spp. based on size [38]. Fecal samples with egg counts > 200 were chosen, and the eggs were collected using a modified rinsing and sieving method adapted from Castro et al. [9]. Sugar gradients were then used for separating the eggs from the fecal debris and the eggs of other parasites (by egg density), using a modification of the method by David and Lindquist [39]. Briefly, the feces were weighed in 25–50 g aliquots and left to soak in tap water until they had a slurry-like consistency (15–20 min). Then, the slurry was poured over a stack of sieves in the mesh size order of 500, 212, 71, and 20 μm (initially, a 38 μm sieve was included, but this was found to decrease the collection of eggs). Starting from the top, the debris in each sieve was washed for 1–2 min, repeated three times, and then discarded, only keeping the debris left in the 20 μm sieve. Next, the 20 μm sieve was washed with deionized water, and the debris was collected in a 75 mL beaker. The debris was left to sediment at 6 °C for 15–20 min; thereafter, the supernatant was removed with a vacuum pump. To isolate the eggs from the eggs of other parasite species and to further reduce the fecal particles, the remaining debris was added to sugar gradients to separate the materials according to density. Three sugar gradients with 20%, 30%, and 40% sucrose were made by dissolving sucrose (Sigma-Aldrich, Stellenborsch, Germany) in boiling water. The gradients were kept at 5 °C until use and left to acclimate to room temperature before use. A Pasteur pipette was placed in a 50 mL centrifugation tube to allow 10 mL of each gradient to be layered in the tube. The 20% gradient was added first, followed by the 30% and 40% gradient, so that the heavier layer would push the less dense layer(s) upwards. The sieved egg sample (0% gradient) was added to the top of the 20% gradient and the tubes were then immediately centrifuged at 2400 g RCF for 7 min. After centrifugation, the eggs were collected between the 0% gradient and the 20% gradient. The eggs were transferred to a clean 20 μm sieve and rinsed thoroughly with deionized water. The eggs from each aliquot of fecal sample were then pooled and stored at 5 °C until use.

### 2.3. Egg Hatch Assay

The egg hatch assay (EHA) method was modified from that of Coles et al. [40]. Approximately, 100 eggs were transferred into individual wells of a 96-well flat-bottom plate. The negative and positive controls were water or either levamisole or albendazole (50 μg/mL), respectively. Moreover, as some of the plant extracts were not water-soluble, we also tested DMSO as 1% of the total fluid (150 μL) to test the eggs’ tolerance to the compound. After the addition of the test compounds, the plates were incubated at 22 °C in a humidified environment. The activity of the compounds was assessed after 48 h of incubation, under light microscopy, whereby the eggs and first-stage larvae were counted.

### 2.4. Larval Development Assay

The larval development assay (LDA) was adapted to this parasite after the modified LDA of Williams et al. [34]. The egg suspension (containing 100 eggs) was transferred into the wells of a 96-well flat-bottom plate. The eggs were added to a 100 μL suspension of deionized water, 15% nutritive media (1% yeast extract suspended in Hank’s balanced salt solution (Sigma-Aldrich, Schellendorf, Germany), antibiotics (300 U/mL penicillin and streptomycin), and antimycotics (10 μg/mL amphotericin B). The negative and positive controls contained deionized water, 1% DMSO, and levamisole (50 μg/mL). The treatments were screened in six replicates in the same plate. The plates were incubated at 22 °C in a humidified environment until the L3 were developed within the negative control wells (around 7–14 days). Determination of the larval stage was then assessed by microscopy.

### 2.5. Larval Migration Assay

The larval migration assay (LMA) was used for assessing the activity of third-stage larvae (L3). The method was modified from that of Williams et al. [34]. The larvae were exsheathed by adding 20 μL of sodium hypochlorite (Sigma-Aldrich, Schellendorf, Germany) pr. mL of L3 suspension; then, they were washed in sterile water. The L3 were then suspended in warm culture media (RPMI-1640 supplemented with L-glutamine (2 mM) and 1% streptomycin and penicillin, Sigma-Aldrich, Schellendorf, Germany) at a concentration of 1 L3/μL. Approximately, 100 larvae were added to each well of a 96-well flat-bottom plate. The positive control consisted of 50 μg/mL levamisole. The plate was then incubated in a humidified environment at 37 °C and 5% CO_2_. Following overnight incubation, 100 μL liquid agar (1.6% in water) was added to each well and slowly mixed with the content of the well. The agar was allowed to set until it formed a gel. Next, 100 μL of RPMI-1640 (with 1% penicillin and streptomycin) was added onto the top of the solidified agar. The assay was then incubated at 37 °C in 5% CO_2_ overnight in a humidified environment. In total, the larvae were incubated for 48 h. The larvae visible on top of the agar were then counted by light microscopy. Inhibition of the larval migration in dose-response experiments was calculated using the following equation:

Relative migration% = 100 − (migrated larvae/mean migration of larvae in control) × 100(1)

### 2.6. Statistical Analyses

All data from the EHA and LMAs were analyzed using GraphPad Prism (9.3.1; GraphPad Software, San Diego, CA, USA). Analysis was performed using one-way ANOVA along with Dunnett’s multiple comparisons test. Differences of *p* < 0.05 were considered significant.

## 3. Results

**Figure 1:**
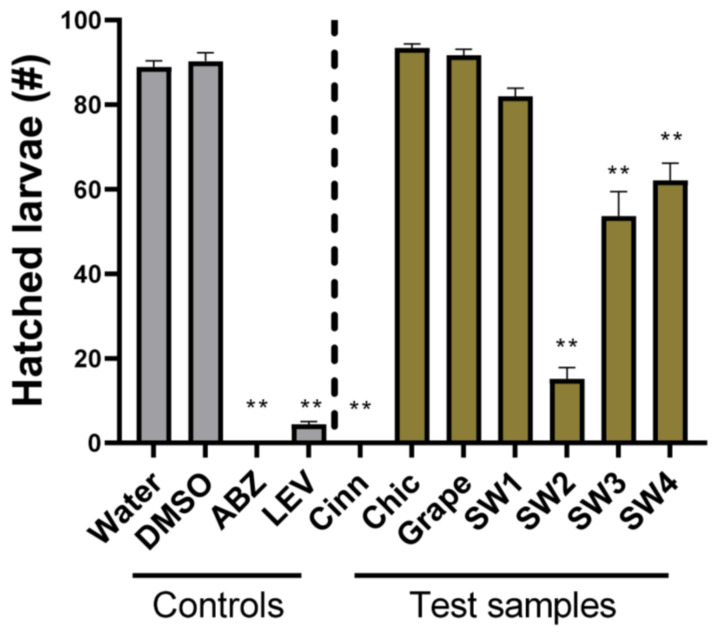
Egg hatch assays with *Uncinaria stenocephala* eggs—eggs were incubated with either water, 1% DMSO, albendazole (ABZ) or levamisole (LEV)—both 50 µg/mL, trans-cinnamaldehyde (Cinn; 10 µg/mL), chicory extract (Chic; 1 mg/mL), grape seed extract (Grape; 1 mg/mL), or 4 different *Saccharina latissima* extracts (SW1-SW4; see Materials and Methods for description; 1 mg/mL). Hatched eggs were counted after 48 h. Data are presented as mean ± S.E.M. (*n* = 6 for each treatment). ** *p* < 0.01 by ANOVA.

As a first step in elucidating the anti-parasitic activity of natural compounds against *U. stenocephala*, we adapted a widely used EHA to this parasite. In the presence of only water or 1% DMSO (negative controls), around 90% of the eggs hatched. In contrast, the anthelmintic drugs albendazole and levamisole suppressed egg hatching to 0% and <5%, respectively (Figure 1). The EHA was thus considered robust enough to assess the activity of the novel anti-parasitic compounds, and the six plant-derived compounds or extracts were evaluated using this assay. Strikingly, cinnamaldehyde completely abolished egg hatching, as seen with albendazole. In contrast, chicory and grape seed extracts had no activity (Figure 1). The seaweed samples displayed variable activity based on the source location and extraction solvent. SW1, derived from Denmark and extracted from methanol/water, showed little capacity to inhibit egg hatching whilst the corresponding sample (SW2) sourced from the Faroe Islands strongly inhibited hatching (*p* < 0.05). The two samples derived from dichrolomethane and methanol (SW3 and SW4) also significantly inhibited hatching (*p* < 0.05), but not to the same extent as SW2. Overall, these data show that cinnamaldehyde and seaweed-derived compounds display anti-parasitic activity against the egg stage of *U. stenocephala*.

**Figure 2:**
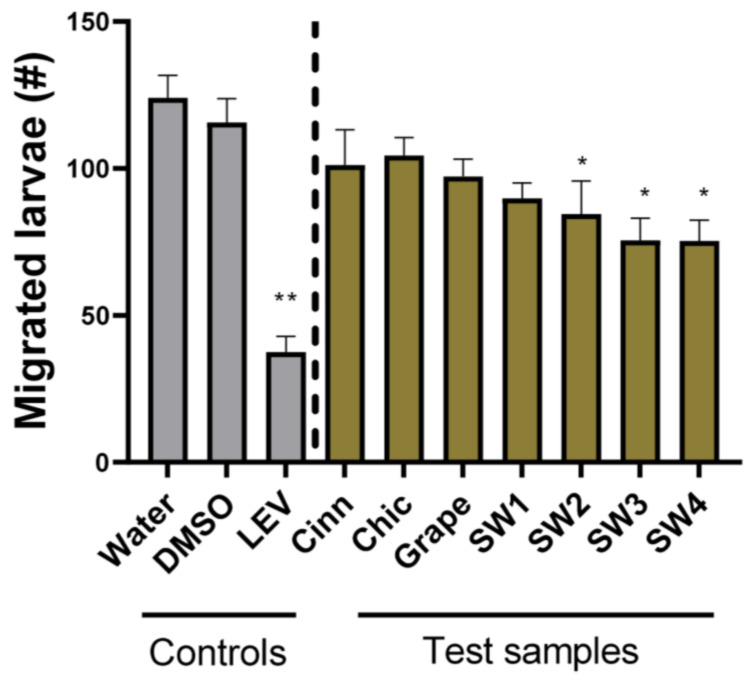
Larval migration assays with *Uncinaria stenocephala* larvae—larvae were incubated with culture media together with either water, 1% DMSO, levamisole (50 µg/mL), trans-cinnamaldehyde (Cinn; 10 µg/mL), chicory extract (Chic; 1 mg/mL), Grape seed extract (Grape; 1 mg/mL), or 4 different *Saccharina latissima* extracts (SW1-SW4; see Materials and Methods for description; 1 mg/mL). Migrated larvae were counted after 24 h incubation. Data are presented as mean ± S.E.M. (*n* = 9 for each treatment). * *p* < 0.05; ** *p* < 0.01 by ANOVA.

We subsequently assessed the ability of the samples to exert anti-parasitic activity against the larval stages. First, we attempted to measure the inhibition of larval development using the LDA, an assay widely used for the assessment of anthelmintic drugs against helminths with free-living larval stages. However, we found the LDA to be unsuitable as we observed only a very small percentage of larvae in our negative control wells developing to the L3 stage, despite repeated attempts (data not shown). In contrast, we previously observed that close to 100% of the larvae developed to the L3 stage when using this assay procedure with other parasites [25,34]. Therefore, we concluded that this LDA was not appropriate for assessing the activity of anthelmintic agents against *U. stenocephala*. Thus, an agar-based LMA that has proven to be a repeatable and robust tool for assessing the activity of anthelmintic compounds against *Ascaris suum* and *Oesophagostomum dentatum* [32,34] was selected for further adaptation. This assay also has the advantage that it can be used to assess activity against in vivo parasitic stages, and thus, it may be the most relevant assay to use. We found that the LMA also produced robust results with *U. stenocephala*. The exsheathed larvae incubated with only water or 1% DMSO migrated in high numbers, whilst incubation with levamisole significantly reduced this migratory ability (*p* < 0.01; Figure 2). Consistent with the EHA, neither grape seed nor chicory extracts reduced larval migration. Interestingly, cinnamaldehyde did not lead to the inhibition of larval migration in the LMA, despite having strong activity in the EHA, (Figure 2). In contrast, three of the four seaweed extracts (SW2-4) did reduce migration (*p* < 0.05)—these were the same samples that exhibited activity in the EHA.

**Figure 3:**
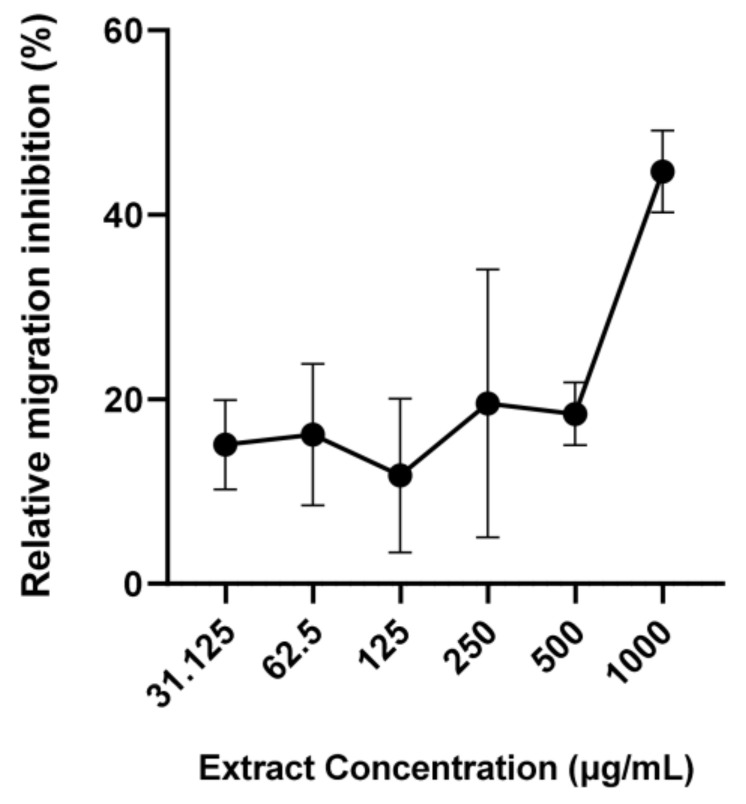
Larval migration assays with different doses of *Saccharina latissima* extract—*Uncinaria stenocephala* third stage larvae were incubated with different doses of a dichrolomethane-methanol extract of *S. latissima* sourced from Grenå, Denmark. Migrated larvae were counted after 24 h incubation. Data are presented as mean ± S.E.M. (*n* = 3 for each treatment).

Based on the combined results of the EHA and LMA, SW3 was chosen for the dose-response LMA experiments. These demonstrated that SW3 exhibited modest but significant dose-response anti-parasitic activity (Figure 3), with close to 50% migration at a concentration of 1 mg/mL, with the activity plateauing at concentrations of ≤ 125 µg/mL.

**Figure 4:**
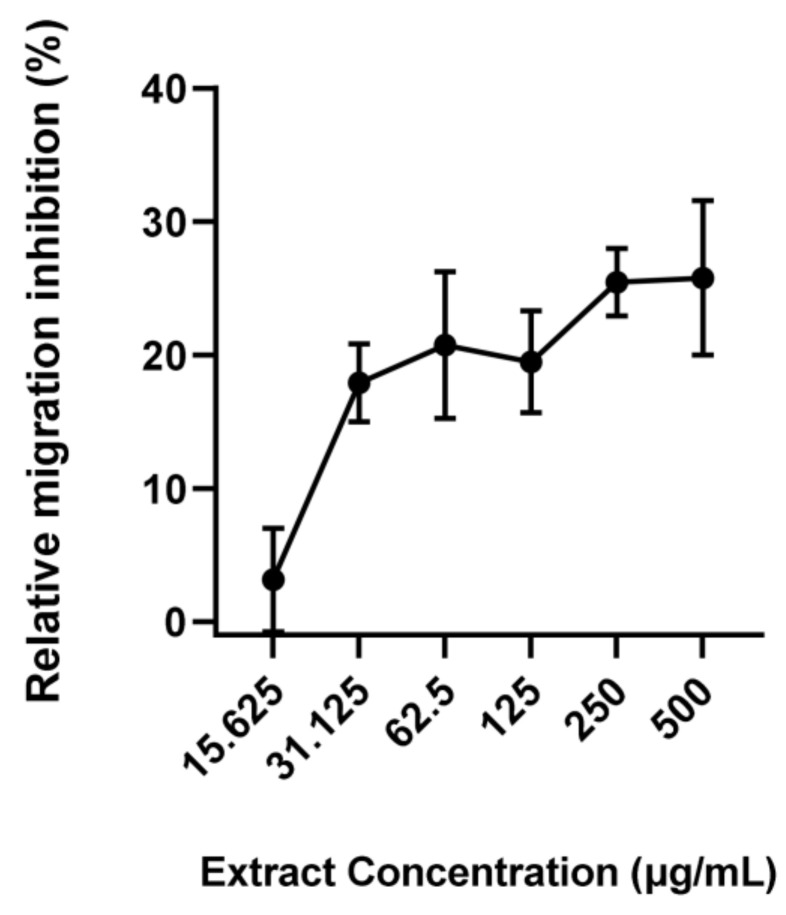
Larval migration assays with different doses of α-linolenic acid—*Uncinaria stenocephala* third stage larvae were incubated with different doses of α-linolenic acid. Migrated larvae were counted after 24 h incubation. Data are presented as mean ± S.E.M. (*n* = 4 for each treatment).

Previously, using a combination of molecular networking and bio-guided fractionation, we identified that a group of fatty acids were the active compounds in *S. latissima* against the swine helminth *A. suum*, with α-linolenic acid (ALA) being the most potent [24]. To confirm whether ALA acid was also active against *U. stenocephala*, we repeated the LMA with the purified fatty acid, and it also demonstrated dose-dependent inhibition of larval migration (Figure 4), providing further evidence that these compounds in seaweed have broad-spectrum, anti-parasitic activity.

## 4. Discussion

In the present study, we demonstrated that seaweed extracts and ALA have inhibitory effects on the larval stages of the *U. stenocephala*. More specifically, the extracts from *S. latissima* were the only samples amongst those we tested (Table 1) to show inhibitory effects on both egg hatching ability and L3 migratory ability. The non-polar SW3 and SW4 extracts caused the most consistent effect as both extracts showed a moderate inhibition on both the egg and the L3. However, the effects were somewhat less pronounced (36.1% and 35.9% in the LMA) than in the previous work by Bonde et al. with *A. suum* L3 (97.8% and 42.1%) [24]. The difference in activity between the polar extracts SW1 and SW2 emphasizes the potential effects that harvesting location and time of year contribute to differences in bioactivity [27].

Bonde et al. [24] examined the compounds of the exact same non-polar extract used in the present study (SW3) and concluded that the most abundant compounds were poly-unsaturated fatty acids (PUFA). These authors found that ALA seemed to possess particularly high anthelmintic activity. When the pure ALA was tested in the present study, it showed a moderate dose-dependent inhibitory effect on L3 and was comparable to the response to the SW3. Therefore, it is likely that this compound is at least partly responsible for the anthelmintic activity of the SW3. Furthermore, Bonde et al. found that a synergistic effect between ALA, stearidonic, and eicosapentaenoic acid caused a high mortality in *A. suum* L3 [24]. This might explain why the ALAs on their own had lower activity than that of SW3 in the present study.

There were contrasting effects of cinnamaldehyde against eggs and L3. Cinnamaldehyde had potent activity against the eggs as no eggs had hatched after 48 h. This corresponds well with the findings by Katiki et al. [26] and Boyko and Brygadyrenok [41]. However, these two studies measured the death of the eggs and embryonated larvae, whereas in the present study only whether the eggs hatched or not was considered. Cinnamaldehyde had a low inhibitory effect on the migratory ability of the L3, which is in contrast to the findings of Williams et al. [42] against *A. suum*. However, as this compound was only tested in a single concentration, it is possible that it could have had an increased effect with increased concentration as it did cause a significant inhibition.

Condensed tannins derived from grape seed and chicory extracts did not have a significant effect on either the eggs or the L3 of this parasite. This contrasts with the previous research, which showed a high activity of these substances against several other parasite species [43]. Whilst the reasons why *U. stenocephala* appears to be resistant to these compounds are not yet clear, our results highlight the diverging activity of natural compounds against different parasites and the importance of empirically testing these novel compounds against the appropriate target species.

Our results suggest that seaweed may have potential anti-parasitic activity when included as a functional feed component in canine diets. Previous studies have included seaweeds in dog foods for different potentially beneficial effects [44,45,46,47]. Pinna et al. found that ingestion of intact seaweeds at an inclusion level of 15 g/kg diet was well tolerated by the dogs and did not alter the apparent total tract nutrient digestibility in their study [47]. It seems that the active compounds of seaweed that induce an anti-parasitic effect are mainly the omega 3 fatty acids [24]. A previous study found that algal extracts containing eicosapentaenoic and docosahexaenoic acid up to an inclusion level of 3% of the diet were safe for both adult dogs and puppies [46]. However, although the general toxicity of seaweed might be low, the effect of inclusion levels needs to be examined further. This also refers to the palatability of the diet when seaweed compounds are included as a functional ingredient in dog foods. Inclusion of *Ascophyllum nodosum* at a low level (0.3% of diet) did not reduce feed intake when added to an extruded kibble diet, but a high inclusion (1% of diet) did lower feed intake, suggesting that inclusion of seaweeds might have to be limited [48]. The putative identification of ALA as an active compound also opens up the possibility that this or related compounds (e.g., fatty acids derived from fish or flaxseed oils) could be purified and administered as encapsulated therapeutics.

In conclusion, we developed robust assays for assessing the activity of different drugs and natural products against the larvae of *U. stenocephala*. The identification of seaweed as a plant with natural anti-parasitic activity should facilitate the development of novel dietary supplements to help control helminth infection in dogs.

## Figures and Tables

**Table 1:** Plant extracts and pure compounds and their concentrations tested in egg hatch assay (EHA) and larval migration inhibition assay (LMIA).

Sample	Source	Concentration
trans-cinnamaldehyde	Sigma-Aldrich	10 µg/mL
Chicory extract	[36]	1 mg/mL
Grape seed extract	[35]	1 mg/mL
Seaweed SW1	[24]	1 mg/mL
Seaweed SW2 *	[24]	≤1 mg/mL
Seaweed SW3	[24]	1 mg/mL
Seaweed SW4	[24]	1 mg/mL
α-linolenic acid **	Sigma-Aldrich	≤500 µg/mL

* Subsequently tested at concentration range in LMIA ** Tested at concentration range—only tested in LMIA.

## Data Availability

All data are contained within the article.
